# Intestinal Obstructions

**Published:** 1871-12

**Authors:** Robert Battey

**Affiliations:** Rome, Ga.


THE GEORGIA

MEDICAL COMPANION:

A MONTHLY ADVISER.

VOL. I. ATLANTA, GA., DECEMBER, 1871. NO. 12

### PART I.

### Original Communications and Special Selections.

### INTESTINAL OBSTRUCTIONS.

BY ROBERT BATTEY, M. D., ROME, GA.

*Case 1st.* Patsy, colored woman, age about 26, married, mother of several children; was attacked with violent colicky-pains in the bowels, nausea and vomiting, in the latter part of December, 1867. During the succeeding four or six weeks, she was attended, rather irregularly, by two or three physicians, besides sundry wise women of her color. In this time, she had several bottles of castor oil, several large doses of calomel, numerous doses of purgative pills, besides, more than one hundred enemata, of varied composition, given with an eight-ounce pewter syringe, in the hands of of the negro nurses; but never more than one syringeful at a time.

Occasionally small liquid evacuations were obtained from the bowels, but the colicy-pains still persisted, and were often violent. Borborygmi frequent and loud. She was now put upon small doses of calomel, carried to moderate ptyalism: opium freely given to control pain, and the system supported with liquid nutriment, as well as the oft-recurring vomiting would admit, until the case terminated, fatally, Februrary 26th.

Post-mortem examination, on the following day, showed no signs of peritonitis, but much engorgement of the intestinal capillaries. The canal was comparatively empty, and greatly inflated with gas throughout. At the lower portion of the sigmoid flexure of the colon, was an intussusception scarcely eight inches from the anus. At the point of obstruction the canal permitted the passage of an ordinary female catheter, but not of the finger; the tissue was much weakened by inflammation and in a semi-gangrenous state. The liver showed numerous deposits of tubercle of considerable size. The womb contained a healthy ovum of about eight weeks.

*Case 2d.* Jimmie F., aged ten years, was taken with colicky-pains in the bowels, on the 27th February, 1868. His mother gave him a full dose of castor-oil with turpentine, which, failing to operate, was repeated in the afternoon. Vomiting came on, with severe paroxysms of pain in the bowels, and persisted through the night. When first seen in the latter part of the night, he was suffering greatly with abdominal pain, frequent vomiting, no evacuation of the bowels, abdomen hard and knotted, facial expression pinched, and expressive of much distress. A full anodyne injection of laudanum was thrown into the rectum, an emolient poultice applied to the abdomen, and preparations began for a *distensile enema.*

After the lapse of an hour, the patient being well under the influence of the laudanum, the distensile enema was administered, consisting of simple warm water, at blood-heat, slowly but perseveringly thrown into the bowels through a Mattson's soft rubber syringe, the water being well compressed around the pipe by the hands of an assistant, until probably *twelve pints* of fluid had passed into the intestines, when he was allowed to get up. This large bulk of fluid was evacuated in installments, through the space of three hours, with much fecal matter, and the case entirely relieved. The little patient was up at play the following evening, and required nothing more.

*Cased 3d.* Julia, five years old, was attacked with symptoms of colic, had some brandy and paragoric, followed by repeated purgatives, with no other results, but to increase the abdominal pain, and excite vomiting. A careful examination of the abdomen revealed a circumscribed hardness in the region of the ileo-cecal valve, which was the seat of much tenderness, as well as pain. There was a peculiarly anxious and pinched expression of the face. A copious distensile enema soon evacuated the bowels, and fully solved the case.

*Case 4th.* Mr. S., 55 years old, attacked with symptoms of colic, took whisky, peppermint, and red pepper-tea, repeated doses of castor-oil and cathartic pills, and applied hot pepper-fomentations to the abdomen, without relief. He was seen on the 26th June, 1868. There was hardness, tenderness and pain in the course of the ascending colon; markedly anxious expression of countenance, pulse quick and rather small, skin bathed in cool perspiration, frequently recurring attacks of billious vomiting.

Preparations were at once made for a distensile enema, which was administered secumdum artem, to the extent of nearly three gallons. After evacuation of the water, all pain ceased; a mild purgative was administered and operated well.

*Case 5th.* Bishop Blanque was siezed with colic from eating watermellon, vomited the contents of the stomach, took brandy and laudanum, followed by purgative dose of calomel and rhubarb. After some hours, the purgative having failed, the abdominal pains increased in violence, with repeated billious vomitings, he was seen. The anxious expression, usually marked in such cases, was observable, the abdomen tender, contracted and hard. A full dose of morphine was administered, followed in an hour by a distensile enema, of moderate bulk, which evacuated the bowels fully, and left nothing to be desired for the comfort of the patient.

*Case 6th.* Sweetie, an interesting little girl of three and a-half years, was seen in the summer of 1867. She had been nearly three weeks without evacuation of the bowels. The abdomen was much distended, tender to the touch, and markedly hard all along the course of the colon, from the cecum to the sigmoid flexure. There was much complaint of atrocious pains in the bowels, which had persisted with increasing severity for a week or more. The rectum was found closely impacted with hard scybalous masses, which were broken down with the terminal joint of the little finger, and removed as completely as could be done. A moderate distensile enema was now administered, and the abdomen carefully kneaded with the fingers over the colon, to break up the masses and encourage them downwards into the rectum. The latter was again emptied as before, the distensile enema and kneading repeated, to bring forward additional installments, until a satisfactory clearance was at length effected, when a dose of castor-oil completed the cure.

*Case 7th.* Mrs. C., 65 years old, had been suffering for some time with a severe diarrhœa, which, yielding to treatment, was followed by obstinate constipation, and this neglected went on to intestinal impaction. She was seen in October, 1868. There was great complaint of intestinal pains, the abdomen was sore to the touch, and a well-defined cylindrical hardness all along the course of the descending and part of the transverse colon. Per verginam the rectum was ascertained to be fully distended by a hard impacted mass of scybalæ.

The index finger *in the vagina* was brought to bear, through the rectovaginal septum upon the fecal mass, breaking it up into fragments, which were successively pushed through the sphincter into a napkin, until the rectum was empty. A distensile enema was then administered; followed by kneading the colon, until a fresh installment was brought down into the rectum, and pushed out by the finger in the vagina as before; and so on until all, or nearly all had been removed, when a mild purgative completed the process.

*Case 8th.* Martin, a negro man, was attacked with colic, from eating fruit, for which he took whisky, and repeated purgatives, without avail. On the second day vomiting supervened, and purgatives were plied again, all to no useful purpose. On the third day he was seen; put under the influence of opium; a distensile enema given; then a dose of castor-oil, and he was promptly up at his work.

*Remarks.*—In the first case cited above, it was manifest, at the post-mortem, that the early use of a distensile enema, of even three pints of warm water, would have rescued this patient *promptly* and *certainly.* This inference was irresistable—as it was the use of purgatives, and purgative enemata in the earlier periods of the illness was continued, with a patient pertinacity worthy of a happier result. Repeated partial evacuations were obtained, to encourage perseverance in the method adopted, and yet as the event proved all to no good end. Why was this so?

In postmortem inspections, after intestinal obstruction, it is not unfrequently observed that restricted areas of both the lesser and larger intestines, are found to be violently and permanently contracted down, into a sort of cord by spasm of the muscular coat, while adjoining portions above and below are of normal or even abnormal distension. It seems quite reasonable to infer that this spasmodic stricturing of the canal is coincident with the severe colicky pains present in the history of the case. While this state of firm contraction exists at any point in the canal, with gaseous distension above and below; if violent peristaltric movements be induced by the exhibition of an active purgative, how readily would the contracted portion slip down and become invaginated in an adjoining dilated part and intussusception be produced.

A similar accident may likewise occur when the exciting cause is an irritating or purgative enema producing the active peristaltic movement.

Again, suppose at some point in the colon a mass of scybala becomes impacted; irritation of the intestine results; colicky pains comes on with flatulent distension, while the muscular coat is strongly contracted and firmly grasping the mass in its fold, as in cases 5, 6, and 7. Imagine the action of a vigorous purgative in such condition, throwing the intestines into violent peristaltic commotion; how easily might a distended portion above reduplicate itself over that below, holding the mass in its grasp. Suppose, now, the three layers overlying the obstructing material by their three-fold muscular power, should expell it to a point lower down, and leave an intussusception behind. In this case the fecal matters might easily be brought away by purgatives or purgative enemata, while the invaginated intestine is by the same agency being more and more firmly grasped in muscular spasm, and the fate of the poor sufferer effectually sealed.

And yet again; suppose a knuckle of intestine has slipped beneath an adventitious band, left by some former local peritonitis and become strangulated; the irritation, the pain, the intestinal muscular spasm which ensues may equally give rise to fatal intussusception, under the stimulus of an active purgative dose.

Has it been the fortune of the reader to have passed his earlier life upon a Georgia plantation? Has it been his lot to witness in person, in its annually recurring season, the various stages and manipulations of the complex art, popularly known by the term "hog-killing," by which the various tissues and organs of that animal are prepared for domestic uses and human sustenance? Has he ever stood over the large tubs of clear spring water, containing the "sausage skins," as they are sometimes called, and observed an old negress, who ordinarily conducts the cleansing process, fish up with her finger the end of an intestinal tube, insert a joint of reed into its calibre, and inflate it with her breath? If so, he has taken his first lesson in the management of intestinal obstruction. See how the dead mass of confused and shapeless membranes progressively assumes form and semblance as the air distends their parieties. How they suddenly become reanimated as it were, and rapidly disentangled from the chaotic confusion, wind themselves, like the serpents of Laocoon, in graceful curves about the sable arm of the operator. Observe more closely, as the breath is blown in, and note the propelling power of the inflation; how it causes the intestine to withdraw itself as though instinctive with real life, from an entanglement aptly comparable to the Gordian knot, with all the ease and grace that a serpent emerges from the tangled grass. Take up a loop of the intestine in the hand, confine it between the fingers, and note with what ready power the distending inflation withdraws it from your grasp. Surely the power of *distension from below* is the very force needed for the relief of intestinal obstruction, whatever be its cause, if indeed the cause be removable by human art.

The requisite distension, for the purpose in view, may be obtained by either of three methods, namely: first, by forcing air into the bowels with a bellows or pump; secondly, by the disengagement of carbonic acid gas in the bowels, from the union of tartaric acid and bicarbonate of soda, separately introduced; and thirdly, by the forcible injection of suitable fluids.

To the first of these, it may well be objected that the introduction of atmospheric air into the bowels produces irritation; that irritation induces peristaltic movements, muscular contractions, muscular spasm, all of which tend towards intussusception, and antagonize the efforts at distension. To the second the same objections apply with yet greater force; and the power invoked, is eminently uncertain in its degree and difficult to be regulated and controlled. On the other hand, to the distensile enema of simple tepid water, no valid objection can be urged. It is eminently unirritating, soothing and relaxing even in its properties. It exerts a force directly proportioned to the bulk employed by reason of the incompressibility of water, a force which can be regulated at will. It is always at hand, and can be successfully administered, if need be, with a hog's bladder and joint of reed.

In *every case* of intestinal obstruction, either feared, suspected, or known to exist, where the duration does not raise a well grounded apprehension of gangrene, an anodyne having been premised; the distensile enema ought to be the *first*, and for the most part need be the *only* power invoked for the cure. In the treatment of colic and fecal impaction, it is wise to abstain from the use of active purgatives until all spasm of the intestines is allayed, and fecal accumulations are removed by the distensile enema.

